# When Prostate Cancer Mimics Multiple Myeloma: Diffuse Bone Marrow Infiltration Revealing Metastatic Prostate Adenocarcinoma With Neuroendocrine Differentiation

**DOI:** 10.7759/cureus.113169

**Published:** 2026-07-22

**Authors:** Latifa Lamine, Chaymae Senoussi, Lamia Nadif, Soundous Bennour, Rania Mokfi, Naoufal N Berrada, Ichrak Bouhou, lazrak Meryam, Farah Boutaagount, Meryem Maskrout, Ghizlane Rais

**Affiliations:** 1 Department of Medical Oncology, University Hospital Center Souss Massa, Faculty of Medicine and Pharmacy, Ibn Zohr University, Agadir, MAR

**Keywords:** aggressive variant prostate cancer, bone marrow aspiration, bone marrow infiltration, bone scintigraphy, metastatic prostate cancer, multiple myeloma mimic, neuroendocrine differentiation, pancytopenia, prostate adenocarcinoma, prostate-specific antigen

## Abstract

Diffuse bone marrow infiltration is an uncommon manifestation of metastatic prostate adenocarcinoma and may mimic hematologic malignancies such as multiple myeloma, resulting in diagnostic delay. Neuroendocrine differentiation further complicates the clinical presentation because of its aggressive biological behavior and atypical metastatic pattern. We report a rare case of metastatic prostate adenocarcinoma with neuroendocrine differentiation revealed by diffuse bone marrow infiltration.

A 59-year-old man presented with diffuse bone pain and pancytopenia and was initially admitted to the hematology department because of suspected multiple myeloma. Bone marrow examination revealed infiltration by malignant epithelial cells, prompting referral for oncological evaluation. Further investigations demonstrated high-grade metastatic prostate adenocarcinoma with neuroendocrine differentiation associated with extensive osteomedullary involvement. The patient received an intensified therapeutic strategy combining androgen deprivation therapy, abiraterone acetate, and platinum-based chemotherapy with docetaxel and carboplatin. This case highlights the importance of considering metastatic prostate cancer in the differential diagnosis of unexplained cytopenia and diffuse bone lesions in male patients. It also emphasizes the diagnostic value of bone marrow examination and the need to recognize aggressive neuroendocrine variants requiring adapted therapeutic strategies.

## Introduction

Prostate cancer is the second most frequently diagnosed malignancy and one of the leading causes of cancer-related death among men worldwide, accounting for more than 1.4 million new cases annually [1,2]. Histologically, conventional acinar adenocarcinoma represents more than 95% of prostate cancers and is characterized by androgen-dependent growth with a marked propensity for skeletal dissemination, the bone being the most frequent site of distant metastasis [3].

Although bone metastases occur in approximately 70-90% of patients with advanced prostate cancer, diffuse bone marrow infiltration remains an uncommon manifestation despite the high frequency of skeletal involvement. It is generally observed in advanced disease and may present with hematological abnormalities, including anemia, thrombocytopenia, or pancytopenia, sometimes closely mimicking primary hematological malignancies such as multiple myeloma and leading to diagnostic delay [4,5].

Among the different histological variants, neuroendocrine differentiation represents a rare but highly aggressive biological subtype that may arise de novo or develop during tumor progression. It is associated with rapid disease progression, atypical metastatic dissemination, poor prognosis, and relative resistance to conventional androgen deprivation therapy [6,7]. Recognition of this entity is clinically important because it frequently exhibits biological and clinical characteristics distinct from those of conventional prostatic adenocarcinoma, often requiring a different therapeutic approach.

We report a rare case of high-volume metastatic prostate adenocarcinoma with neuroendocrine differentiation revealed by diffuse bone marrow infiltration and extensive osteomedullary metastases, initially suspected to be multiple myeloma. This case highlights the importance of considering metastatic prostate cancer in the differential diagnosis of unexplained cytopenia associated with diffuse bone lesions. It also emphasizes that recognizing neuroendocrine differentiation has important prognostic and therapeutic implications, as these tumors often exhibit aggressive biological behavior and may require treatment strategies beyond conventional androgen deprivation therapy.

## Case presentation

A 59-year-old man with a history of chronic tobacco smoking and no other significant medical history presented with progressive right-sided sciatalgia associated with diffuse bone pain. The symptoms had initially appeared approximately four years before presentation as intermittent pain and gradually worsened over time, becoming persistent and refractory to analgesic treatment. During the months preceding diagnosis, the patient also reported occasional lower urinary tract symptoms, including dysuria and pollakiuria. During the six months preceding diagnosis, the clinical course was marked by progressive gait impairment and deterioration of the patient's general condition, including asthenia, anorexia, and unintentional weight loss.

Because of persistent diffuse bone pain associated with abnormal blood counts, the patient was initially admitted to the hematology department with a suspected diagnosis of multiple myeloma. Initial laboratory investigations revealed severe anemia, thrombocytopenia, and mild neutropenia. Baseline hematological findings are summarized in Table 1.

**Table 1:** Baseline and follow-up hematological parameters.

Parameter	At diagnosis (December 2025)	Latest follow-up (July 2026)	Reference range
Hemoglobin (g/dL)	8.0	11.9	13.0-17.0
White blood cell count (×10⁹/L)	4.2	6.0	4.0-10.0
Absolute neutrophil count (×10⁹/L)	1.47	1.96	2.0-7.5
Absolute lymphocyte count (×10⁹/L)	1.78	2.20	1.0-4.0
Platelet count (×10⁹/L)	66	236	150-400

Bone marrow aspiration was therefore performed and demonstrated diffuse infiltration of the bone marrow by cohesive clusters of malignant epithelial cells, findings suggestive of metastatic carcinoma rather than a primary hematological malignancy. The patient was subsequently referred to the medical oncology department for further investigation of the primary tumor.

On admission to our department, the patient had an Eastern Cooperative Oncology Group (ECOG) performance status of 2. Neurological examination revealed asymmetric motor weakness predominantly involving the right lower limb, raising concern for spinal cord compression. Digital rectal examination demonstrated a stony-hard, irregular prostate highly suspicious for malignancy. No palpable peripheral lymphadenopathy was identified.

**Figure 1:**
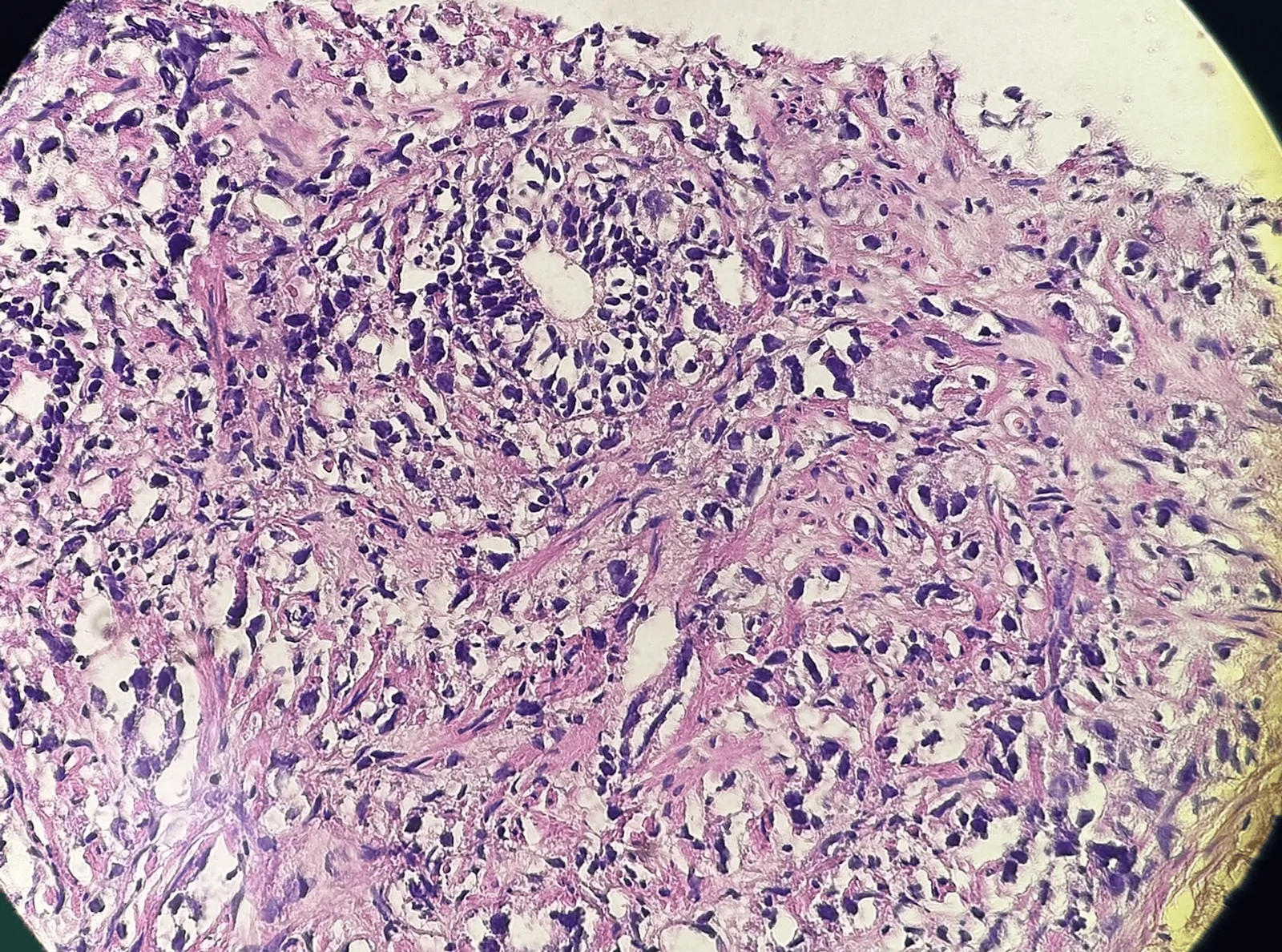
Histopathological examination of the prostate biopsy demonstrating high-grade conventional acinar adenocarcinoma (H&E stain, ×200).

Serum prostate-specific antigen (PSA) was elevated to 71.65 ng/mL. In the context of the patient's extensive osteomedullary metastatic involvement, the PSA level appeared lower than might be expected for the overall disease burden. Although this finding is not specific, when considered together with the aggressive clinical presentation and subsequent immunohistochemical findings, it contributed to the suspicion of an associated neuroendocrine component. Transrectal prostate biopsy revealed high-grade conventional acinar adenocarcinoma involving approximately 90% of the biopsy material, with a Gleason score of 9 (5+4), corresponding to International Society of Urological Pathology (ISUP) grade group 5 (Figure 1).

**Figure 2:**
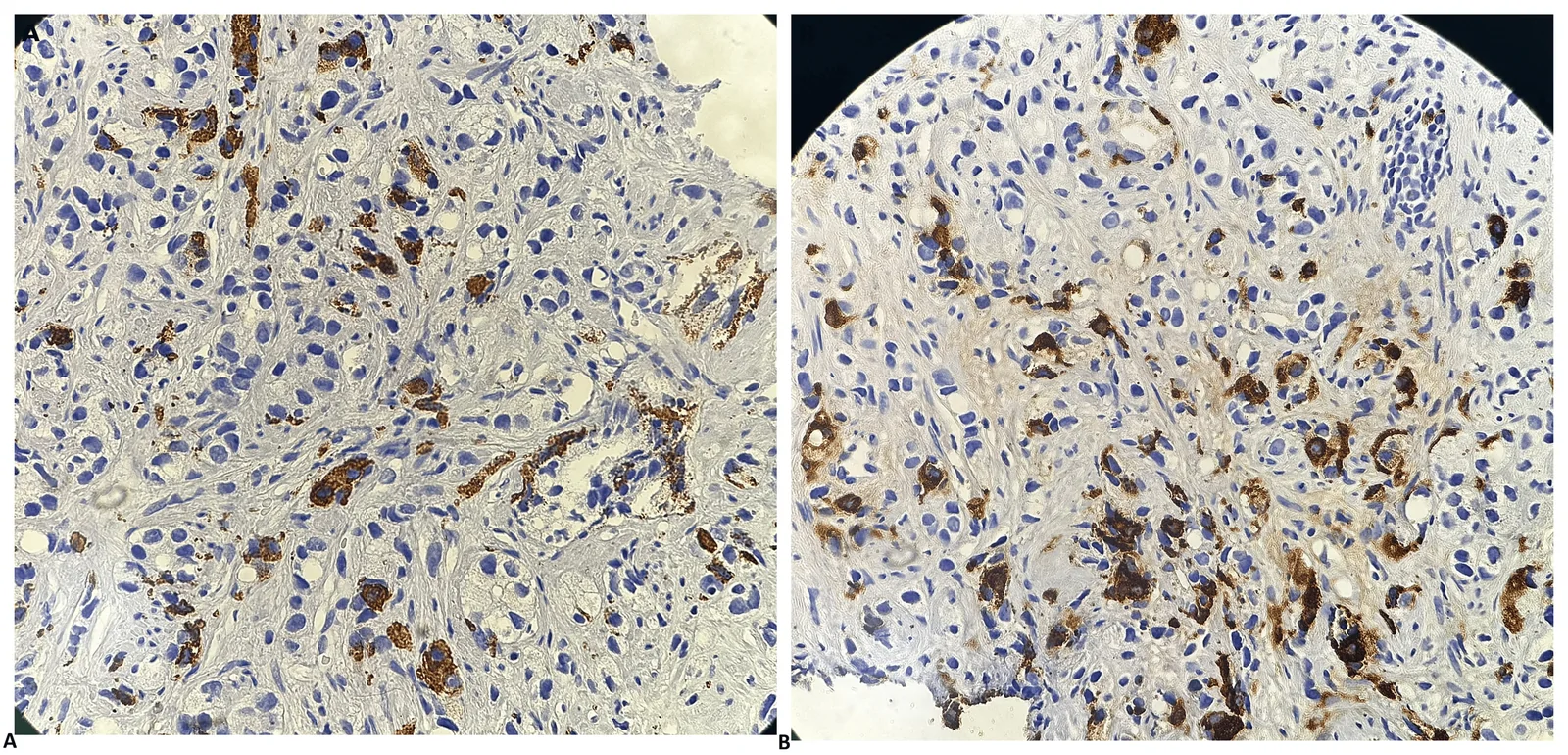
Immunohistochemical staining demonstrating neuroendocrine differentiation. (A) Positive immunostaining for chromogranin A involving approximately 25% of tumor cells. (B) Synaptophysin positivity confirming the neuroendocrine component (immunohistochemistry {IHC}, ×200).

The diagnosis of prostatic adenocarcinoma was established on histomorphological examination. Given the unusually aggressive clinical presentation and the marked discordance between the serum PSA level and the metastatic tumor burden, additional immunohistochemical studies were performed to investigate a possible neuroendocrine component. Immunohistochemistry demonstrated positive staining for chromogranin A and synaptophysin in approximately 25% of tumor cells, focal positivity for CD56, and a Ki-67 proliferation index of approximately 50%, confirming an associated neuroendocrine component involving approximately 25% of the tumor (Figures 2A, 2B).

**Figure 3:**
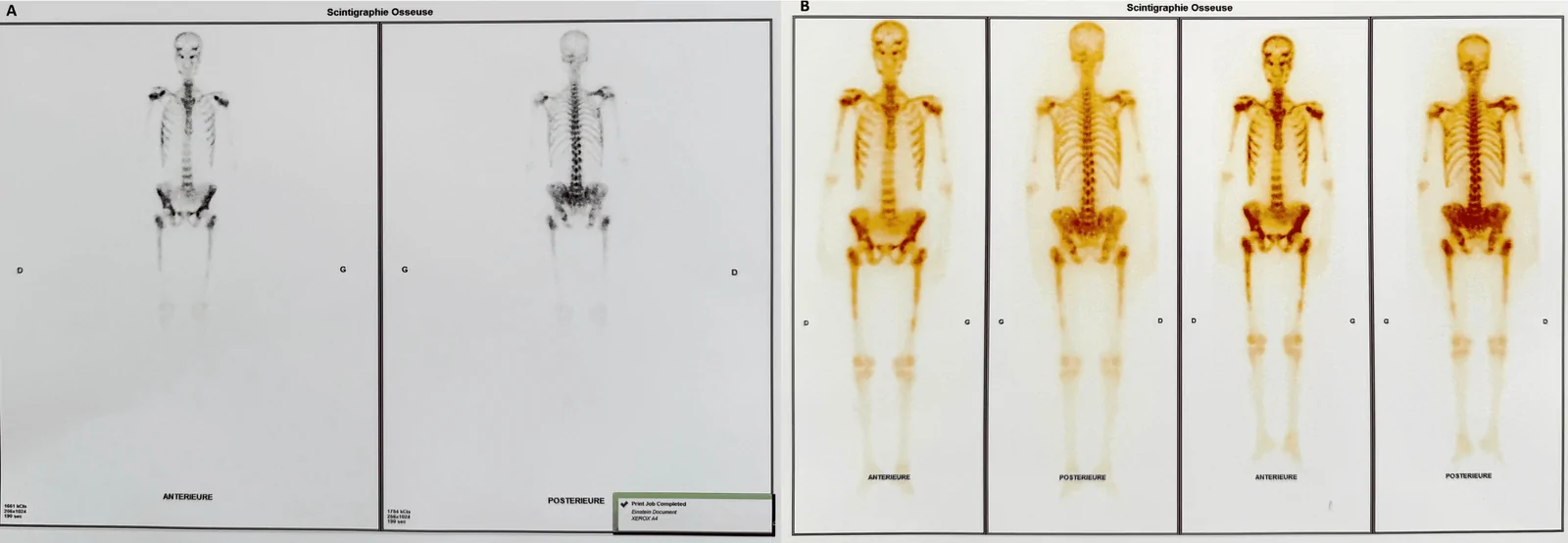
Whole-body bone scintigraphy demonstrating a metastatic super scan. (A) Anterior and posterior planar scintigraphic images. (B) Color-rendered scintigraphic images.

Given the neurological findings, whole-spine magnetic resonance imaging (MRI) was performed to exclude metastatic spinal cord compression and demonstrated no evidence of epidural extension or spinal cord involvement. For disease staging, contrast-enhanced computed tomography (CT) of the chest, abdomen, and pelvis revealed diffuse osteosclerotic lesions involving both the axial and appendicular skeleton without evidence of visceral metastases. Whole-body bone scintigraphy demonstrated the classic appearance of a metastatic super scan, characterized by diffuse intense skeletal radiotracer uptake with markedly reduced renal visualization, consistent with extensive osteomedullary metastatic involvement (Figures 3A, 3B).

The case was discussed at a multidisciplinary tumor board meeting. Considering the high-volume metastatic disease, Gleason score 9, neuroendocrine differentiation, diffuse bone marrow infiltration, and the discordance between PSA level and tumor burden, an individualized intensified therapeutic strategy was considered appropriate. The patient received triplet systemic therapy consisting of androgen deprivation therapy (ADT), next-generation hormonal therapy with abiraterone acetate plus prednisone, and platinum-based chemotherapy combining docetaxel and carboplatin, to address both the conventional adenocarcinoma and the associated neuroendocrine component.

The patient demonstrated a favorable early clinical and biological response to treatment. His general condition progressively improved, with marked reduction of bone pain and recovery of functional capacity. Hematological abnormalities resolved completely, with normalization of both anemia and thrombocytopenia, reflecting recovery from bone marrow infiltration. Serum PSA showed a marked decline from 71.65 ng/mL at diagnosis (December 2025) to 2.09 ng/mL at the seven-month follow-up (July 2026), consistent with an excellent early response to the intensified triplet therapeutic strategy. At the same follow-up evaluation, the patient maintained sustained clinical improvement, continued normalization of hematological parameters, and showed no evidence of disease progression.

## Discussion

In the present case, the association of diffuse bone pain and cytopenias initially suggested multiple myeloma. Similar diagnostic challenges have been described by Noor et al. and Yigit and Geyer, in whom metastatic prostate adenocarcinoma involving the bone marrow initially mimicked hematologic malignancies because of unexplained cytopenias and atypical bone marrow findings [4,5]. In all three cases, bone marrow examination played a pivotal role in establishing the diagnosis after hematologic disorders had initially been suspected. Together, these reports and our case highlight the importance of considering metastatic prostate cancer in elderly men presenting with diffuse bone pain and otherwise unexplained cytopenias.

Another remarkable aspect of our case is the presence of neuroendocrine differentiation. Neuroendocrine prostate cancer represents a rare but highly aggressive biological entity associated with rapid disease progression, atypical metastatic dissemination, and poor clinical outcomes [6-9]. Neuroendocrine differentiation may occur de novo or emerge during disease evolution, particularly after prolonged androgen receptor pathway inhibition. Focal neuroendocrine differentiation has been described in a subset of advanced prostate adenocarcinomas, whereas pure neuroendocrine carcinoma remains uncommon [8,9].

In our patient, the diagnosis was established by the coexistence of conventional acinar adenocarcinoma and positive immunohistochemical staining for chromogranin A, synaptophysin, and CD56, consistent with adenocarcinoma with neuroendocrine differentiation [8-10]. Although prostate-specific immunohistochemical markers such as prostate-specific antigen (PSA), NKX3.1, and AMACR were not available, the diagnosis of prostatic origin was established based on characteristic histomorphology, markedly elevated serum PSA level, clinical presentation, and prostate biopsy findings. Nevertheless, the absence of additional prostate-specific immunohistochemical markers represents a limitation of this report.

Neuroendocrine differentiation is frequently associated with reduced androgen receptor signaling and may result in PSA levels that appear relatively low compared with the overall disease burden [8,9]. Although PSA concentrations vary considerably among patients with metastatic prostate cancer, discordance between PSA level and metastatic extent should prompt consideration of neuroendocrine differentiation when supported by compatible clinical, radiological, and pathological findings. In our patient, the PSA concentration of 71.65 ng/mL was interpreted in conjunction with extensive osteomedullary metastatic involvement and the immunohistochemical findings rather than as an isolated diagnostic feature.

Imaging also contributed substantially to disease characterization. Conventional imaging with computed tomography (CT) of the chest, abdomen, and pelvis demonstrated extensive osteosclerotic skeletal metastases. Whole-body bone scintigraphy demonstrated the classic appearance of a metastatic super scan, characterized by diffuse skeletal tracer uptake and markedly reduced renal visualization, consistent with extensive osteomedullary metastatic involvement. More recently, prostate-specific membrane antigen (PSMA) positron emission tomography/computed tomography (PET/CT) has demonstrated superior sensitivity for metastatic staging of prostate cancer [11]. However, reduced PSMA expression has been reported in neuroendocrine variants, potentially limiting its diagnostic performance. In such aggressive tumors, fluorodeoxyglucose (FDG)-PET/CT may provide complementary information because of the increased metabolic activity of neuroendocrine lesions [12].

Therapeutic management of aggressive prostate cancers with neuroendocrine differentiation remains challenging because prospective evidence is limited. Although treatment intensification with androgen deprivation therapy combined with next-generation androgen receptor pathway inhibitors and/or docetaxel has become the standard of care for many patients with metastatic hormone-sensitive prostate cancer, evidence supporting these strategies in tumors with neuroendocrine differentiation remains limited [13,14]. Previously reported cases of metastatic prostate cancer with diffuse bone marrow involvement generally received androgen deprivation therapy alone or conventional docetaxel-based regimens [4,5]. In contrast, our patient presented with several adverse clinicopathological features, including Gleason score 9, corresponding to International Society of Urological Pathology (ISUP) grade group 5, diffuse osteomedullary infiltration, immunohistochemically confirmed neuroendocrine differentiation, and extensive skeletal metastatic disease [15]. After multidisciplinary discussion, an individualized treatment strategy combining androgen deprivation therapy, abiraterone acetate, and platinum-based chemotherapy with docetaxel plus carboplatin was selected. This therapeutic approach was selected based on the patient's aggressive clinicopathological characteristics and the available evidence supporting the use of platinum-based chemotherapy in selected patients with aggressive variant prostate cancer rather than as an established standard-of-care recommendation [7,16].

Although platinum-based chemotherapy has shown encouraging activity in aggressive variant prostate cancer with neuroendocrine features, evidence remains limited, and no standardized treatment strategy has been established for mixed adenocarcinoma with neuroendocrine differentiation [7,16].

Overall, this case highlights several important clinical messages. Metastatic prostate cancer should remain in the differential diagnosis of unexplained pancytopenia associated with diffuse bone pain in men, particularly when bone marrow examination demonstrates epithelial infiltration. Discordance between PSA levels and metastatic burden should prompt consideration of neuroendocrine differentiation, especially when supported by immunohistochemical findings. Finally, aggressive variants of prostate cancer require individualized multidisciplinary management, and treatment intensification may be considered in carefully selected patients according to their clinicopathological characteristics rather than as a standard therapeutic approach.

## Conclusions

Prostate adenocarcinoma with neuroendocrine differentiation and diffuse bone marrow infiltration is a rare and aggressive clinical entity that may initially mimic hematologic malignancies, particularly in patients presenting with cytopenias and diffuse bone pain. This case highlights the importance of considering metastatic prostate cancer in the differential diagnosis of unexplained cytopenias associated with diffuse osteomedullary lesions and underscores the essential role of bone marrow examination, histopathology, and immunohistochemistry in establishing an accurate diagnosis and identifying aggressive tumor variants. Finally, discordance between PSA levels and metastatic tumor burden should raise suspicion of neuroendocrine differentiation when interpreted in the appropriate clinicopathological context. Early recognition of this aggressive phenotype and individualized multidisciplinary management may contribute to favorable clinical, hematological, and biochemical responses in carefully selected patients, although further studies are needed to better define the optimal therapeutic strategy.
